# Clinical Comparison of COVID Waves 2–5. An Inpatient Retrospective Comparative Analysis From Karachi, Pakistan

**DOI:** 10.1093/ofid/ofaf072

**Published:** 2025-02-07

**Authors:** Muneeba Ahsan Sayeed, Elisha Shalim, Shaiza Farman, Fizza Farooqui, Beenish Syed, Ishfaque Ahmed, Anika Iqbal, Aneel Kumar, Raniyah Akhter, Furkan Hyder, Hasan Ali Shah, Adeel Hussain, Sarwat Rasheed, Saba Afshan, Rizwana Salik Nukrich, Madiha Raza, Haseeb U Rehman, Abdul Razzaque Memon, Abdul Wahid Rajput, Muhammad Saeed Quraishy

**Affiliations:** Infectious Diseases Department, Sindh Infectious Diseases Hospital & Research Center, Dow University of Health Sciences, Karachi, Pakistan; Research Department, Sindh Infectious Diseases Hospital & Research Center, Dow University of Health Sciences, Karachi, Pakistan; Research Department, Sindh Infectious Diseases Hospital & Research Center, Dow University of Health Sciences, Karachi, Pakistan; Microbiology Department, Sindh Infectious Diseases Hospital & Research Center, Dow University of Health Sciences, Karachi, Pakistan; Infectious Diseases Department, Sindh Infectious Diseases Hospital & Research Center, Dow University of Health Sciences, Karachi, Pakistan; Infectious Diseases Department, Sindh Infectious Diseases Hospital & Research Center, Dow University of Health Sciences, Karachi, Pakistan; Infectious Diseases Department, Sindh Infectious Diseases Hospital & Research Center, Dow University of Health Sciences, Karachi, Pakistan; Pulmonology Department, Sindh Infectious Diseases Hospital & Research Center, Dow University of Health Sciences, Karachi, Pakistan; Pulmonology Department, Sindh Infectious Diseases Hospital & Research Center, Dow University of Health Sciences, Karachi, Pakistan; Anaesthesiology Department, Sindh Infectious Diseases Hospital & Research Center, Dow University of Health Sciences, Karachi, Pakistan; Anaesthesiology Department, Sindh Infectious Diseases Hospital & Research Center, Dow University of Health Sciences, Karachi, Pakistan; Anaesthesiology Department, Sindh Infectious Diseases Hospital & Research Center, Dow University of Health Sciences, Karachi, Pakistan; Critical Care Department, Sindh Infectious Diseases Hospital & Research Center, Dow University of Health Sciences, Karachi, Pakistan; Pulmonology Department, Sindh Infectious Diseases Hospital & Research Center, Dow University of Health Sciences, Karachi, Pakistan; Pulmonology Department, Sindh Infectious Diseases Hospital & Research Center, Dow University of Health Sciences, Karachi, Pakistan; Anaesthesiology Department, Sindh Infectious Diseases Hospital & Research Center, Dow University of Health Sciences, Karachi, Pakistan; Pulmonology Department, Sindh Infectious Diseases Hospital & Research Center, Dow University of Health Sciences, Karachi, Pakistan; Sindh Infectious Diseases Hospital & Research Center, Dow University of Health Sciences, Karachi, Pakistan; Sindh Infectious Diseases Hospital & Research Center, Dow University of Health Sciences, Karachi, Pakistan; Dow University of Health Sciences, Karachi, Pakistan

**Keywords:** comparison, COVID, disease severity, outcome, waves

## Abstract

**Background:**

Each coronavirus disease 2019 (COVID-19) wave is unique in its clinical presentation and outcome. In this study, we compared the clinical characteristics and outcomes of COVID waves 2–5 in inpatient settings.

**Methods:**

A retrospective study was conducted at the Sindh Infectious Diseases Hospital and Research Center on adult patients who were admitted with a positive COVID polymerase chain reaction from July 2020 to March 2022. SPSS 26 was used to analyze the data.

**Results:**

A total of 3190 COVID-19 patients were admitted. Wave 2 had the highest percentage of discharges compared with mortality (81%; *P* = .0001). Cytokine release syndrome was most common in wave 3 (32.7%; *P* = .0001). Severe COVID on admission was predominant in wave 4 (79.4%; *P* = .0001), with the highest rates of intubation (27.1%; *P* = .0001), septic shock (24.3%; *P* = .0001), and disease progression (50.8%; *P* = .0001). In wave 5, the majority were elderly (median age, 68 years) and had mild COVID (22.4%; *P* = .0001), most had comorbidities (84.6%; *P* = .0001), and the ratio of acute kidney injury was high (29.2%; *P* = .0001). Mortality was lowest in wave 2 (18.9%; *P* = .0001) and highest in wave 4 (42.5%; *P* = .0001; odds ratio, 3.18; 95% CI, 2.6–3.8; compared with wave 2).

**Conclusions:**

Each wave had some unique characteristics compared with other waves, with wave 4, driven by the Delta variant, being the deadliest one in terms of disease severity and outcomes.

Severe acute respiratory syndrome coronavirus 2 (SARS-CoV-2) was announced as a global pandemic and termed coronavirus disease 2019 (COVID-19) by the World Health Organization (WHO) in March 2020 [1]. SARS-CoV-2 has been reported as a Beta variant of coronavirus, which is related to Middle East respiratory syndrome coronavirus and SARS-CoV [2]. The pathogenicity of SARS-CoV-2 infection is that it enters human cells and binds to ACE2 receptors in various organs of the human body [3]. Based on WHO data, >7 million people have died due to COVID-19 [4].

Wave 1 of COVID occurred in Pakistan from March to July 2020 [5, 6]. Later COVID was managed efficiently in Pakistan, which prevented the spread of infection by timely lockdowns, social distancing, rapid diagnostic testing, and building up of infrastructure for patient care and hospitalization [6, 7]. According to the government of Pakistan, wave 2 began on October 28, 2020, when daily cases reached up to 750, and lasted until January 2021 [5]. Wave 3 started in March 2021 and lasted until May 2021; during wave 3, a new variant, B.1.1.7, of COVID-19 emerged and was reported to be associated with an increased risk of death [5, 8]. Wave 4 in Pakistan of the Delta variant (B.1.617.2) occurred from July 2021 to September 2021 [5]. During wave 4, there were >1 245 000 total confirmed cases, with ∼3000 new cases recorded daily in the country. In wave 5 of COVID-19, the first Omicron (B.1.1.529) case was identified in November 2021, and the positivity rate increased to >40% by January 28, 2022 [5]. Ahmad et al. conducted a national analysis on the 5 COVID waves to address the impact of COVID-19 in Pakistan and reported that each wave followed the characteristic pattern of a complete infectious diseases epidemic. It was interesting to see that the duration from the onset of a wave to its peak became progressively shorter, while the interval from the wave peak to trough progressively became longer [9]. In Pakistan, wave 1 was the longest wave; it lasted for 150 days, while wave 5 was the shortest, lasting for 83 days due to increased testing and implementation of smart lockdown [9]. At a national level in Pakistan, the highest oxygen bed–admission ratio was in wave 4, while the highest ventilator utilization ratio was in wave 3 [9].

The COVID vaccination process was initiated in Pakistan in February 2021, toward the end of wave 2. It initially catered to health care workers and gradually expanded to involve the elderly and the general population in a stepwise manner. More than half of the eligible population was fully vaccinated by wave 5 [9]. To date, Pakistan has witnessed 6 COVID-19 waves, with a death toll of >30 000 [10].

The most common clinical manifestations were cough, fever, and dyspnea leading to pneumonia in all COVID-19 waves [11]. Comorbidities played an important role in disease progression and mortality, including chronic illness [12, 13], obesity, and advanced age [13, 14]. Buschner et al., on comparing the 5 COVID waves in Bavaria, reported that individuals who died from COVID-19 were significantly more likely to be obese or to have comorbidities like renal insufficiency, chronic lower respiratory disease, diabetes mellitus, and, except during the third wave, dementia or degenerative nervous system diseases [15]. However, certainly other factors may have contributed to increased mortality. During wave 1 of COVID-19, limited clinical experience and scarcity of data were huge challenges. Later the emergence of therapeutic options and vaccination, especially after wave 2, helped in combating this disease. However, viral factors like the emergence of virulent variants continued to pose treatment challenges. With the emergence of new vaccines and therapies globally, the rate of mortality declined significantly [16, 17]. These advances played a pivotal role in improving survival [18–22]. De Paepe et al. compared 3 COVID waves in Belgium and reported that mortality declined with each successive wave, despite the rising severity of the disease and the higher number of patients with comorbidities [23]. Noriali et al. on comparing COVID waves reported that pneumonia was significantly less prevalent in the Omicron wave compared with the previous wave due to higher vaccination rates [24]. Due to these host, viral, and social factors, we have observed variations in outcomes as well as in the clinical spectrum of COVID patients in different COVID waves [5]. Therefore, the aim of this study was to compare the clinical characteristics, disease severity, and outcomes of hospitalized COVID-19 patients who were admitted from the second to fifth COVID-19 waves at a specialized COVID-19 center in Karachi, Pakistan.

## METHODS

This was a single-center retrospective study that included 3190 COVID patients who were admitted at Sindh Infectious Diseases Hospital & Research Center (SIDH & RC), a 175-bed dedicated infectious diseases hospital in Karachi, Pakistan, from July 2020 to March 2022. All male and female patients who were >16 years of age and tested positive for COVID on nasopharyngeal/oropharyngeal swab polymerase chain reaction were included. Patients who were <16 years of age, did not receive primary treatment at SIDH, or had a hospital stay of <24 hours were excluded.

All confirmed cases of COVID-19 were included in the study from July 2020 to March 2022. Demographic data, disease category, history of preexisting conditions that can increase the risk factor, vaccination status, disease progression, disease severity, intensive care unit (ICU) stay, laboratory parameters, and outcome were collected from the hospital medical records and Hospital Management Information System (HMIS) and recorded in clinical research form. Comorbidities were identified through self-reporting, which was then verified by reviewing prior medical records or conducting confirmation tests, as well as through diagnoses made during admission. To minimize the issue of missing data, multiple sources were utilized, including patient files, flowsheets, registries, and the HMIS. Additionally, national COVID-19 surveillance efforts prompted hospital administration to maintain accurate medical records, resulting in a very low percentage of missing data. Patients with incomplete data were excluded from the study. The primary author and research associates collected the data. The data were managed and handled in Excel before the analysis started. The patient data included a clear diagnosis by a physician or by medical record and a history of COVID.

COVID waves were divided using the following definitions:

Wave 2: Data were collected from October 2020 to January 2021.
Wave 3: Data were collected from March 2021 to May 2021.
Wave 4: Data were collected from July 2021 to September 2021.
Wave 5: Data were collected from November 2021 to March 2022.

Classification of COVID-19 severity was based on the national COVID-19 treatment guidelines in effect at the time. Mild COVID-19 was characterized by symptoms such as fever, cough, sore throat, fatigue, headache, myalgia, nausea, vomiting, diarrhea, and loss of taste or smell without shortness of breath and with normal oxygen saturation (SpO2) on room air. Moderate COVID-19 was defined by an SpO2 of ≤94% but >90%, accompanied by mild pneumonia. Severe COVID-19 was characterized by an SpO2 of ≤90% on room air with >50% lung infiltrates visible on a chest radiograph. Critical COVID-19 involved acute respiratory distress syndrome (ARDS), multiorgan dysfunction, or septic shock, with the patient requiring noninvasive or invasive ventilation.

The data included in the study were summarized and presented using tables and graphs. The Kruskal-Wallis test was used to check the normality of the data. Medians and interquartile ranges were used to present the non–normally distributed data of continuous variables. Numbers and percentages were used for categorical data. The Fisher and chi-square tests were used for categorical data. *P* < .05 was considered statistically significant. All risk factors with univariate *P* values <.05 were included in multivariate logistic Cox regression analysis to adjust the confounders and to determine the risk factors for overall mortality and in each wave; results were given as odd ratios (ORs) with 95% CIs and as hazard ratio (HRs). In the analysis of COVID waves 4 and 5, binary logistic regression was utilized to identify the factors associated with mortality. A Kaplan-Meier survival curve was plotted for 30-day survival. The analysis was done on Statistical Package for the Social Sciences (SPSS; version 26.0) and Prism GraphPad (version 8).

## RESULTS

### Demographics and Clinical Characteristics

**Figure 1.**
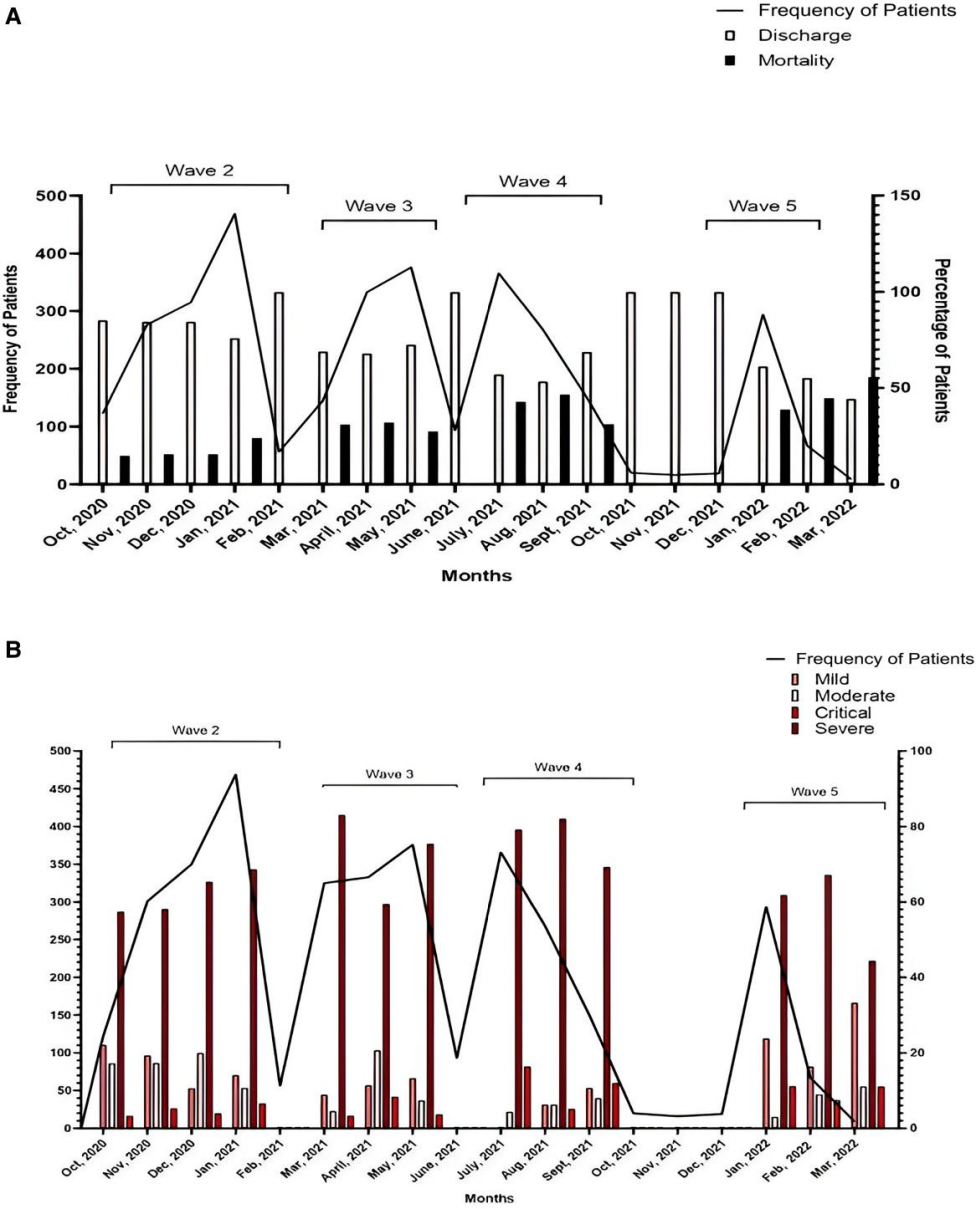
Epi curve of COVID inpatients with outcome (*A*) and disease severity (*B*). Abbreviation: COVID-19, coronavirus disease 2019.

In this study, 3190 patients were included, with a median (interquartile range [IQR]) age of 62 (52–70) years. Patients were then divided into 4 groups based on the period of occurrence of COVID (wave 2, wave 3, wave 4, and wave 5) according to the COVID-19 waves that occurred in Karachi, Pakistan. An epi-curve of hospitalized COVID-19 patients is shown in Figure 1.

Wave 2 included 1182 (37.1%) patients; wave 3 had 810 (25.4%) patients, while waves 4 and 5 had 783 (24.5%) and 415 (13%) patients, respectively. The majority were males (1933, 60.6%) as compared with females (1257, 39.4%). Comorbidities were seen in 2341 (73.4%) patients, in whom hypertension (HTN) was the most common, seen in 1492 (46.8%), followed by diabetes mellitus (1202, 37.7%) and ischemic heart disease (IHD; 369, 11.6%). Of these, 48 (1.5%) patients were smokers. Of the total 3190 patients, 463 (14.5%) had mild, 305 (9.6%) had moderate, and 2213 (69.4%) had severe COVID, while 209 (6.6%) had critical COVID at the time of admission. Mortality was seen in 955 patients, with an overall in-hospital mortality rate of 29.9%. Disease progression was seen in 1382 (43.3%) patients. Only 13.17% of mild patients progressed to moderate or severe COVID; 67.2% of the moderate patients progressed to severe or critical disease, while 28.10% of severe patients progressed to critical COVID. Demographics and clinical characteristics are shown in Table 1.

**Table 1.** Baseline Demographics and Clinical Characteristics of COVID-19 Patients

Parameters	All n = 3190	Wave 2 n = 1182 (37.1%), No. (%)	Wave 3 n = 810 (25.4%), No. (%)	Wave 4 n = 783 (24.5%), No. (%)	Wave 5 n = 415 (13%), No. (%)	*P* Value Overall
Age, median (IQR), y	62 (52–70)	62 (53–70)	60 (50–70)	60 (50–70)	68 (60–76)	<.0001
Male	1933 (60.6)	766 (64.8)	520 (64.2)	417 (53.3)	230 (55.4)	<.0001
Comorbidity	2341 (73.4)	897 (75.9)	556 (68.6)	537 (68.6)	351 (84.6)	<.0001
Diabetes mellitus (DM)	1202 (37.7)	387 (32.7)	331 (40.9)	301 (38.4)	183 (44.1)	<.0001
Hypertension (HTN)	1492 (46.8)	447 (37.8)	401 (49.5)	384 (49)	260 (62.7)	<.0001
Asthma	100 (3.1)	38 (3.2)	21 (2.6)	21 (2.7)	20 (4.8)	.157
Ischemic heart disease (IHD)	369 (11.6)	124 (10.5)	111 (13.7)	62 (7.9)	72 (17.3)	<.0001
Cerebral vascular accident (CVA)	64 (2)	18 (1.5)	13 (1.6)	8 (1)	25 (6)	<.0001
Chronic kidney disease (CKD)	99 (3.1)	34 (2.9)	14 (1.7)	18 (2.3)	33 (8)	<.0001
Malignancy	22 (0.7)	10 (0.8)	2 (0.2)	5 (0.6)	5 (1.2)	.223
Thyroid abnormality	77 (2.4)	21 (1.8)	18 (2.2)	20 (2.6)	18 (4.3)	.033
Chronic obstructive pulmonary disease (COPD)	46 (1.4)	15 (1.3)	13 (1.6)	3 (0.4)	15 (3.6)	<.0001
Others	448 (14)	189 (16)	81 (10)	77 (9.8)	101 (24.3)	<.0001
Smoker	48 (1.5)	12 (1)	12 (1.5)	6 (0.8)	18 (4.3)	<.0001
Disease category on admission						
Mild COVID	463 (14.5)	189 (16)	112 (13.8)	69 (8.8)	93 (22.4)	.0001
Moderate COVID	305 (9.6)	132 (11.2)	109 (13.5)	46 (5.9)	18 (4.3)	
Severe COVID	2213 (69.4)	796 (67.3)	537 (66.3)	622 (79.4)	258 (62.2)	
Critical COVID	209 (6.6)	65 (5.5)	52 (6.4)	46 (5.9)	46 (11.1)	

Abbreviations: COVID-19, coronavirus disease 2019; IQR, interquartile range.

### Comparison of Different COVID Waves (Wave 2–Wave 5)

In comparison with other waves, wave 5 was unique in terms of affected age group, comorbidity status, and mild COVID hospitalization. The patients infected in wave 5 were mostly older, with a median (IQR) age of 68 (60–76) years (*P* < .0001). More than two-thirds of patients in wave 5 had comorbidities (84%; *P* < .0001) compared with waves 2 (75.9%), 3 (68.6%), and 4 (68.6%), with hypertension being the most common one. The highest percentage of hospitalizations of mild COVID patients was 22.4% in wave 5 owing to the other underlying comorbidities compared with waves 2 (16%), 3 (13.8%), and 4 (8.8%).

In terms of disease severity, wave 4 proved to be the most serious wave, having the highest percentage of cases of severe COVID-19 (79.4%; *P* < .0001) on the day of admission compared with waves 2, 3, and 5.

### Comparison of Outcomes of COVID Waves

The median (IQR) hospital stay was 6 (4–11) days; median hospital stay was lowest in wave 5 compared with other waves (5 days; *P* < .0001). In comparison, wave 4 had the highest rates of ICU admissions (42.4%; *P* = .017), mechanical ventilation (27%; *P* < .0001), and requirement for inotropes (28.1%; *P* < .0001). Of the 3190 patients, cytokine release syndrome (CRS) was seen in 919 (28.8%) patients and was predominant in wave 3 (32.7%; *P* < .0001), as shown in Table 2.

**Table 2.** Comparison of COVID Complications and Outcomes in Different COVID Waves

Parameters	All n = 3190, No. (%)	Wave 2 n = 1182 (37.1%), No. (%)	Wave 3 n = 810 (25.4%), No. (%)	Wave 4 n = 783 (24.5%), No. (%)	Wave 5 n = 415 (13%), No. (%)	*P* Value
Time of hospital stay, median (IQR), d	6 (4–11)	7 (4–10)	6 (4–11)	7 (4–13)	5 (3–9)	<.0001
Intensive care unit (ICU) stay	1226 (38.4)	425 (36)	299 (36.9)	332 (42.4)	170 (41)	.017
Mechanical ventilation	637 (20)	204 (17.3)	165 (20.4)	212 (27.1)	56 (13.5)	<.0001
Inotropes	692 (21.7)	199 (16.8)	174 (21.5)	220 (28.1)	99 (23.9)	<.0001
Cytokine release syndrome (CRS)	919 (28.8)	334 (28.3)	265 (32.7)	250 (31.9)	70 (16.9)	<.0001
Complications	1678 (52.6)	523 (44.2)	421 (52)	479 (61.2)	255 (61.4)	<.0001
Adverse respiratory distress syndrome (ARDS)	1098 (34.4)	377 (31.9)	263 (32.5)	327 (41.8)	131 (31.6)	<.0001
Pneumonia	1116 (35)	286 (24.2)	284 (35.1)	372 (47.5)	174 (41.9)	<.0001
Non-ST-elevation myocardial infarction (NSTEMI)	361 (11.3)	101 (8.5)	79 (9.8)	110 (14)	71 (17.1)	<.0001
Septic shock	599 (18.8)	167 (14.1)	166 (20.5)	190 (24.3)	76 (18.3)	<.0001
Pulmonary embolism (PE)	236 (7.4)	69 (5.8)	66 (8.1)	69 (8.8)	32 (7.7)	.065
Acute kidney injury (AKI)	655 (20.5)	171 (14.5)	174 (21.5)	189 (24.1)	121 (29.2)	<.0001
Pneumothorax	12 (1)	7 (0.9)	13 (1.7)	3 (0.7)	35 (1.1)	<.0001
Subcutaneous emphysema	38 (1.2)	18 (1.5)	10 (1.2)	9 (1.1)	1 (0.2)	<.0001
Disease progression	1382 (43.3)	451 (38.2)	347 (42.8)	398 (50.8)	186 (44.8)	<.0001
Discharged	2234 (70.1)	959 (81.1)	571 (70.5%)	450 (57.5)	254 (61.4)	<.0001
Died	955 (29.9)	223 (18.9)	239 (29.5)	333 (42.5)	160 (38.6)	

Abbreviations: COVID-19, coronavirus disease 2019; IQR, interquartile range.

In-hospital complications were seen in 1678 (52.6%) patients; waves 4 and 5 had the highest frequency of COVID-associated complications (61.2% and 61.4%, respectively; *P* < .0001), while frequency of COVID-associated complications was low in waves 2 (44.2%) and 3 (52%). ARDS, pneumonia, septic shock, and pulmonary embolism were predominantly seen in wave 4, while non-ST-elevation myocardial infarction (NSTEMI) and acute kidney injury (AKI) were predominant in wave 5. Disease progression was highest in wave 4 (50.8%; *P* < .0001).

Overall in-hospital mortality was seen in 955 (29.9%) patients and was 18.9% in wave 2, 29.5% in wave 3, 42.5% in wave 4, and 38.6% in wave 5; therefore, it was highest in wave 4 (42.5%; *P* < .0001; OR, 3.18; 95% CI, 2.6–3.8; compared with the second wave) and lowest in wave 2 (18.9%; *P* < .0001).

### Risk Factors and Mortality

The risk factors for mortality were evaluated using a Cox regression model as mentioned in Table 3. The multivariate model showed that the risk of mortality increased with advancing age (age group 56–75 years: HR, 2.3; 95% CI, 1.4–3.6; *P* < .0001; and age >75 years: HR, 3.4; 95% CI, 2.14–5.54; *P* < .0001), septic shock (HR, 1.21; 95% CI, 1.04–1.40; *P* = .011), cytokine release syndrome (HR, 1.23; 95% CI, 1.07–1.40; *P* = .004), and mechanical ventilation (HR, 1.22; 95% CI, 0.99–1.51; *P* = .021), while comorbidities and vaccination status were not associated with overall mortality risk.

**Table 3.** Univariate and Multivariate Analysis to Determine the Risk Factors for Mortality Among Hospitalized COVID Patients

Category	Overall n = 3190			
	Univariate HR (95% CI)	*P* Value	Multivariate aHR (95% CI)	*P* Value
Age	1.025 (1.02–1.03)	<.0001	1.02 (1.02–1.03)	<.0001
Age groups				
16–35 y	1.0		1.0	
36–55 y	1.53 (0.95–2.4)	.079	1.53 (0.95–2.45)	.078
56–75 y	2.3 (1.47–3.7)	.0001	2.3 (1.4–3.6)	<.0001
>75 y	3.5 (2.2–5.5)	.0001	3.4 (2.14–5.54)	<.0001
Male	1.01 (0.88–1.15)	.88		
Female	1.0			
Comorbidities	1.06 (0.92–1.23)	.43		
Diabetes mellitus	1.01 (0.89–1.15)	.934		
Hypertension	1.2 (1.03–1.33)	.014	1.12 (0.89–1.27)	.085
Chronic kidney disease	1.56 (1.12–2.17)	.008	1.35 (0.97–1.9)	.07
Vaccinated	0.88 (0.7–1.1)	.267		
ICU stay	0.95 (0.83–1.08)	.416		
Myocardial infarction	1.23 (1.04–1.47)	.016	1.12 (0.93–1.34)	.211
Septic shock	1.19 (1.03–1.37)	.017	1.21 (1.04–1.40)	.011
Pulmonary embolism	1.0 (0.82–1.23)	.96		
Cytokine release syndrome	1.18 (1.03–1.35)	.014	1.23 (1.07–1.40)	.004
Mechanical ventilation	1.231 (1.08–1.4)	.003	1.22 (0.99–1.51)	.021

Abbreviations: aHR, adjusted hazard ratio; COVID-19, coronavirus disease 2019; HR, hazard ratio; ICU, intensive care unit.

A subgroup analysis within each wave was also done using Cox regression, as shown in Table 4.

**Table 4.** Univariate and Multivariate Analysis to Determine the Risk Factors for Mortality in Individual COVID Waves (Waves 2–5)

	Wave 2 n = 1182				Wave 3 n = 810				Wave 4 n = 783				Wave 5 n = 415			
	Univariate HR (95% CI)	*P* Value	Multivariate aHR (95% CI)	*P* Value	Univariate HR (95% CI)	*P* Value	Multivariate aHR (95% CI)	*P* Value	Univariate HR (95% CI)	*P* Value	Multivariate aHR (95% CI)	*P* Value	Univariate HR (95% CI)	*P* Value	Multivariate aHR (95% CI)	*P* Value
Age	1.0 (1.0–1.02)	.03	1.0 (0.9–1.0)	.13	1.0 (1.0–1.02)	<.0001	1 (1.0–1.04)	.0001	1 (1.0–1.04)	.0001	1.0 (1.0–1.04)	.0001	1.03 (1.01–1.04)	.0001	1.0 (1–1.04)	<.0001
Age groups														.004		
16–35 y	1.0		1.0		1.0		1.0		1.0		1.0		1.0		1.0	
36–55 y	0.5 (0.3–0.9)	.04	0.5 (0.2–1.0)	.05	1.9 (0.6–6.1)	.282	1.8 (0.6–5.8)	.323	2.9 (1.3–6.0)	.013	3.1 (1.4–7.3)	.006	4.5 (0.6–35)	.138	4.4 (0.6–33.0)	.152
56–75 y	0.7 (3.5–1.4)	.29	0.6 (0.3–1.3)	.19	3.5 (1.1–11.0)	.032	3.5 (1.1–11.0)	.033	4.1 (1.8–9.0)	.001	4.4 (1.9–10.0)	.0001	6.1 (0.8–44.0)	.071	6.1 (0.8–43.0)	.073
>75 y	0.9 (0.4–1.8)	.76	0.9 (0.4–1.9)	.86	3.9 (1.2–12.8)	.026	3.9 (1.2–13.0)	.026	6.0 (2.8–15.0)	.0001	6.9 (3.0–16.5)	.0001	10.2 (1.4–73.8)	.021	9.3 (1.3–67.0)	.027
Male	0.9 (0.7–1.3)	.71			1.3 (0.9–1.6)	.094			1 (0.8–1.4)	.711			1.2 (0.9–1.6)	.285		
Female	1.0				1.0				1.0				1.0			
Comorbidities	1.1 (0.8–1.6)	.49			1.1 (0.8–1.5)	.347			1 (0.7–1.3)	.997			1.3 (0.8–1.8)	.278		
Diabetes mellitus	0.9 (0.7–1.2)	.94			1.1 (0.8–1.4)	.49			1 (0.8–1.2)	.997			1.3 (0.9–1.8)	.087		
Hypertension	1.3 (0.9–1.6)	.08			1.2 (0.9–1.6)	.092			1 (0.9–1.4)	.25			1.1 (0.81–1.5)	.506		
Chronic kidney disease	2.2 (1.3–3.8)	.006	1.9 (1.1–3.5)	.02	1.4 (0.6–3.5)	.424			2 (1.3–3.8)	.006	2.3 (1.2–4.8)	.019	0.8 (0.5–1.7)	.733		
Unvaccinated									1.0				1.0			
Vaccinated	-				-				0.8 (0.6–1.1)	.114			0.65 (0.8–1.4)	.652		
ICU stay	2.9 (2.2–3.7)	<.0001	1.4 (0.9–2.2)	.087	1.3 (1.0–1.7)	.044	1.3 (1.0–1.8)	.048	2.0 (1.6–2.0)	.0001	1.8 (1.4–2.3)	.0001	1.7 (1.2–2.3)	.002	1.3 (0.8–1.8)	.248
Myocardial infarction	1.9 (1.4–2.8)	<.0001	1.1 (0.7–1.6)	.587	1.4 (0.9–1.9)	.057	1.7 (1.2–2.4)	.003	1.04 (0.7–1.4)	.806			1.4 (0.92–2.1)	.118		
Septic shock	2.8 (2.2–3.7)	<.0001	1.1 (0.7–1.6)	.751	1.02 (0.7–1.3)	.914			1.2 (0.96–1.6)	.106			1.3 (0.9–1.8)	.216		
Pulmonary embolism	2.1 (1.5–3.1)	<.0001	1.1 (0.7–1.7)	.651	1.5 (1.0–2.2)	.05	1.5 (0.9–2.2)	.053	1.08 (0.7–1.52)	.658			2.7 (1.3–5.5)	.007	2.4 (1.2–4.9)	.02
Cytokine release syndrome	2.1 (1.6–2.7)	<.0001	1.3 (0.9–1.7)	.07	1.6 (1.3–2.2)	<.0001	1.6 (1.3–2.2)	<.0001	1.5 (1.2–1.8)	.001	1.3 (1.02–1.6)	.035	1.6 (1.1–2.5)	.027	1.5 (0.9–2.3)	.11
Mechanical ventilation	3.6 (2.8–4.7)	.0001	2.3 (1.5–3.7)	<.0001	1.0 (0.8–1.4)	.763			1.24 (0.9–1.6)	.079			1.2 (0.7–1.9)	.332		

Abbreviations: aHR, adjusted hazard ratio; COVID-19, coronavirus disease 2019; HR, hazard ratio; ICU, intensive care unit.

Wave 2: Chronic kidney disease (HR, 1.9; 95% CI, 1.1–3.5; *P* = .018), cytokine release syndrome (HR, 1.3; 95% CI, 0.9–1.7; *P* = .07), and mechanical ventilation (HR, 2.3; 95% CI, 1.5–3.7; *P* < .0001) were independent risk factors for mortality.

Wave 3: Advancing age (>56 years: HR, 3.5; 95% CI, 1.1–11; *P* = .033), ICU stay (HR, 1.3; 95% CI, 1.0–1.8; *P* = .048), myocardial infarction (HR, 1.7; 95% CI, 1.2–2.4; *P* = .003), pulmonary embolism (HR, 1.5; 95% CI, 0.9–2.2; *P* = .05), and cytokine release syndrome (HR, 1.6; 95% CI, 1.3–2.2; *P* < .0001) were associated with mortality.

Wave 4: Advancing age (age group 35–55 years: HR, 3.1; 95% CI, 1.4–7.3; *P* = .006; age group 56–75 years: HR, 4.4; 95% CI, 1.9–10; *P* = .0001; age group >75 years: HR, 6.9; 95% CI, 3.0–16.5; *P* = .0001), chronic kidney disease (HR, 2.3; 95% CI, 1.2–4.8; *P* = .019), ICU stay (HR, 1.8; 95% CI, 1.4–2.3; *P* = .0001), and cytokine release syndrome (HR, 1.3; 95% CI, 1.02–1.6; *P* = .035) increased the risk of mortality.

Wave 5: Advancing age (>75 years: HR, 9.3; 95% CI, 1.3–67; *P* = .027) and pulmonary embolism (HR, 2.4; 95% CI, 1.2–4.9; *P* = .02) were risk factors for mortality.

### Disease Severity and Mortality

**Figure 2.**
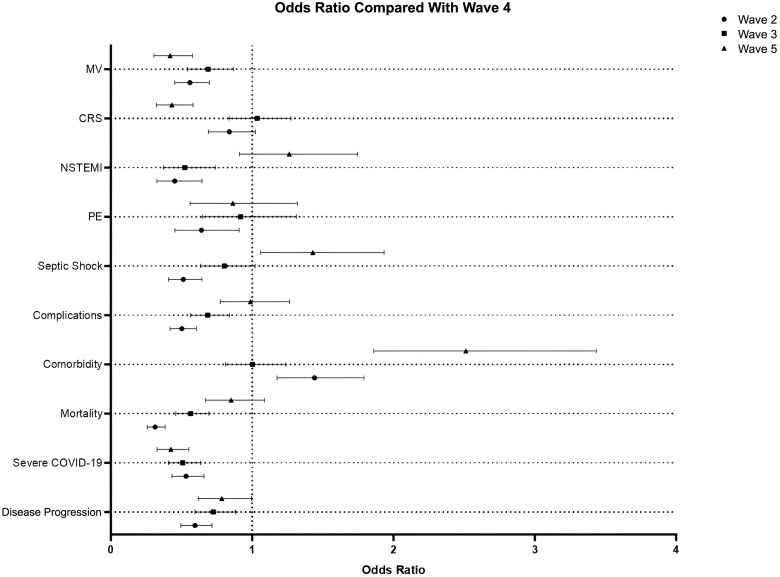
Forest plot to compare COVID wave 4 with other COVID waves. Abbreviations: COVID-19, coronavirus disease 2019; CRS, cytokine release syndrome; MV, mechanical ventilation; NSTEMI, non-ST-elevation myocardial infarction; PE, pulmonary embolism.

Based on disease severity, the overall mortality rate in critical COVID was 67.8%. This rate was 47.6% in wave 2, 76.9% in wave 3, 72.9% in wave 4, and 75% in wave 5. In severe COVID, the overall in-hospital mortality rate was 40.7%; this rate was 40.9% in wave 2, 37.2% in wave 3, 39.7% in wave 4, and 47.4% in wave 5. However, the mortality rate was lower in moderate and mild COVID-19 patients. The overall mortality rate was highest in wave 4. Figure 2 and Supplementary Table 1 compare wave 4 with other waves. Supplementary Table 2 compares the clinical characteristics of survivors of waves 4 and 5 with those of nonsurvivors.

### A**ssociation of Vaccination Status With Mortality and Disease Progression**

Of the 3190 patients, 8.9% of the patients were vaccinated. In wave 4, only 17.75% of the patients were vaccinated, and in that group the mortality rate was lower compared with unvaccinated patients (35% vs 65%; OR, 0.6901; 95% CI, 0.473–1.013; *P* = .058). Moreover, in wave 5, 31.32% of patients were vaccinated and thus had a lower mortality rate compared with unvaccinated patients (32.3% vs 67.7%; OR, 0.6714; 95% CI, 0.422–1.036; *P* = .0821), but these results were not statistically significant on multivariate analysis, as shown in Table 4.

In wave 4, 49.9% were >56 years of age, and when stratified for age mortality in this age group was greater in unvaccinated compared with vaccinated patients (51% vs 38%; *P* = .04).

Moreover, disease progression was greater in unvaccinated patients compared with vaccinated patients in waves 4 and 5 (OR, 1.409; 95% CI, 0.991–2.005; *P* = .05; and OR, 1.421; 95% CI, 0.998–2.023; *P* = .051; respectively).

### Survival Curve Analysis

**Figure 3.**
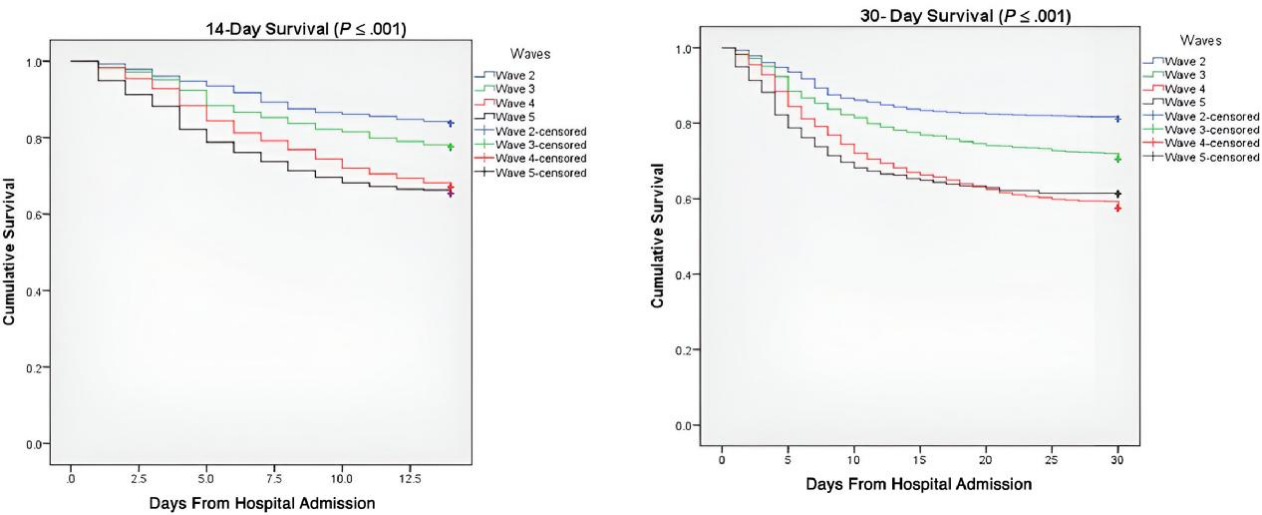
Kaplan-Meier survival estimates of 14- and 30-day mortality of COVID-19 patients admitted during wave 2, wave 3, wave 4, and wave 5. Abbreviation: COVID-19, coronavirus disease 2019.

Wave 2 had the best survival distribution, in which the median (IQR) survival was 22 (17.4–26.5; *P* < .05). This was followed by wave 3 with a median (IQR) survival of 18 (15.8–20.1; *P* < .05). Waves 4 and 5 had the worst survival distribution, with median (IQR) survival of 15 (13.05–16.9; *P* < .05) and 12 (9.4–14.5; *P* < .05), respectively, compared with all other waves, as shown in Figure 3. The 14-day mortality rate was 16.2%, 22.5%, 33.1%, and 34.7% in waves 2, 3, 4, and 5, respectively, while 30-day mortality was 18.4%, 28.3%, 40.9%, and 38.8% in waves 2, 3, 4, and 5, respectively.

## DISCUSSION

SARS-CoV-2 has shown a wide spectrum of disease severity, ranging from mild to critical infection leading to death [25]. The outcome of COVID-19 severe illness is influenced by many factors including age, gender, and preexisting illnesses such as diabetes, hypertension, and cardiovascular diseases. In this study, we compared the disease severity, outcomes, and other clinical characteristics of the COVID-19 waves that occurred in Karachi, Pakistan. The current and previous [26] studies have shown that older patients with COVID-19 infection are more susceptible to severe and critical COVID-19 than middle-aged patients. This age-dependent variability in the severity of COVID-19 could be explained by decreased cell-mediated immune function and reduced humoral immune function [27]. Our study showed that most of the patients who had severe and critical COVID had a median (IQR) age of 60 (50–70) years. Wave 5 had the highest number of elderly patients as compared with other waves, with the second highest mortality. The percentage of males was higher than the that of females in all 4 waves of COVID-19, which is in accordance with previous studies as well as a meta-analysis [28]. Our study reported that hypertension was the most common comorbidity, followed by diabetes mellitus, as seen in other studies [13–16, 29].

The highest number of COVID hospitalizations was seen in wave 2, but the disease progression was less compared with other waves. In the second wave, B.135 (Beta variant) was the predominant variant, as shown in other studies [5, 8]. Though our study does not provide data on the predominant variant, considering the timeline and similar geographical location, it can be assumed that our patients were also mostly infected with the same variant.

In wave 3, most of the admitted patients had severe COVID as compared with wave 2 and had an increased risk of disease progression and death. A new variant, B.1.1.7, of COVID-19 emerged in the country during March–May 2021 and was reported to increase the risk of death compared with wave 2 [5]. Understandably, we had similar findings.

We found that hospitalized patients infected in wave 4 had a significantly higher risk of experiencing severe/critical disease as compared with waves 2, 3, and 5. It was reported that wave 4 of COVID-19 in Pakistan was caused by the Delta variant [5]. It has also been reported that patients infected by the Delta variant have a significant chance to experience moderate or severe/critical disease in the 14-day period. Compared with the Beta variant, the Delta variant is associated with more severe disease and a higher risk of hospitalization, as evident from our data too [30].

In wave 5, most of the hospitalized patients were elderly, with a lesser degree of disease severity as compared with other waves. This low disease severity can be explained either by a higher vaccination rate or by low virulence of the Omicron variant that emerged in this wave. In vitro studies also suggested that the Omicron variant has low efficiency of replication and fusion compared with the Delta variant, which can partly explain its low virulence [30].

In general, we found that advanced age, septic shock, cytokine release syndrome, and mechanical ventilation were independent risk factors for mortality. However, wave-specific factors were different. In wave 2, chronic kidney disease, cytokine release syndrome, and mechanical ventilation were associated with mortality, while in wave 3 age, ICU stay, myocardial infarction, pulmonary embolism, and cytokine release syndrome increased the risk of mortality. In wave 4, age, chronic kidney disease, ICU stay, and cytokine release syndrome and in wave 5 age and pulmonary embolism were risk factors for mortality.

It has been observed that vaccination did not improve outcome until wave 5. Jablonska et al. also reported that vaccination did not decrease the average daily deaths significantly during waves 3 and 4 [31]. Lopez et al. reported the same [32]. However, a notable reduction in hospitalizations and progression of disease to death was observed by the end of wave 4 and throughout wave 5 [33, 34]. We found that vaccination did not affect overall mortality rate on multivariate analysis, but it had a significant impact when stratified for advanced age groups. It also reduced the risk of disease progression in waves 4 and 5.

Our study has several limitations; data were collected retrospectively, leading to potential information bias. As this was a single-center study, the data are not generalizable. In addition, the details of wave 1 are not included in this study as our hospital opened just before wave 2. We were also unable to perform whole-genome sequencing on all the patients to identify variants in different waves and used data from national surveillance and the limited data from our molecular laboratory to label a certain variant for a particular wave [9]. This study has compared neither the socioeconomic implications of COVID-19 nor how structural factors like hospital capacity, resource allocation, and socioeconomic disparities have affected the outcome of COVID-19. Global data have highlighted that structural factors like hospital capacity, availability of medical staff, socioeconomic disparities, and public health infrastructure significantly influenced COVID-19 outcomes during different pandemic waves [35, 36]. Regions with limited hospital resources, including ICU beds and ventilators, faced higher mortality rates. Efficient allocation of resources and infrastructure improvements were key to better outcomes in subsequent COVID waves, highlighting the importance of proper resource allocation for preparedness for future pandemics [35, 36]. Investments in surveillance systems and collection of real-time infrastructure data can enable timely, evidence-based responses that could help in building up a framework on the management of future pandemics and facilitate the conduct of multicenter prospective studies, which generate data in real time. Future studies can be done to look at the effects of long COVID and to understand the long-term effectiveness of COVID vaccines, the therapeutic options, and the effects of socioeconomic factors on outcome.

## CONCLUSIONS

In summary, each COVID wave had certain distinct characteristics compared with the other waves. Wave 2 had the lowest mortality, while wave 4 had the highest mortality. Wave 3 was associated with CRS, while in wave 5 mostly elderly were hospitalized, with a higher number of mild COVID hospitalizations owing to comorbidities. Future studies should be conducted to assess the impact of structural factors and socioeconomic and ethical disparities on outcomes in COVID-19, which will help to promote preparedness for future pandemics.

## Supplementary Material

ofaf072_Supplementary_Data

## Supplementary Data

Supplementary materials are available at *Open Forum Infectious Diseases* online. Consisting of data provided by the authors to benefit the reader, the posted materials are not copyedited and are the sole responsibility of the authors, so questions or comments should be addressed to the corresponding author.

## Notes

***Author contributions.*** Conceptualization, M.A.S; methodology, M.A.S., F.F., and E.S.; software, E.S.; validation, M.A.S., F.F., E.S., T.M., and S.F.; formal analysis, S.F. and E.S.; investigation, B.S., I.A., A.K., R.A., F.H., H.A.S., A.H., and S.R.; data curation, S.A., R.S.N., M.R., and H.R.; writing—original draft preparation, M.A.S., E.S., and F.F.; writing—review and editing, M.A.S., F.F., and E.S.; visualization, M.A.S.; supervision, A.W.R., A.R., and M.S.Q.; and project administration, M.S.Q. All authors have read and agreed to the published version of the manuscript.

***Data availability.*** Data set will be available on request.

***Institutional review board.*** The study was conducted in accordance with the Declaration of Helsinki and approved by the Institutional Review Board (or Ethics Committee) of Dow University of Health Sciences (IRB-3183/DUHS/Approval/2023/434 and date of approval 15-Nov-2023).

***Informed consent.*** Patient consent was waived by the IRB as it is a retrospective review of medical records.

***Financial support.*** This research received no external funding.
