# Supplementary material for: Clinical Comparison of COVID Waves 2–5. An Inpatient Retrospective Comparative Analysis From Karachi, Pakistan
Source: Open Forum Infect Dis. 2025 Feb 7;12(3):ofaf072. doi: 10.1093/ofid/ofaf072 (PMC11890920; doi:10.1093/ofid/ofaf072)
Supplement: ofaf072_Supplementary_Data [file ofaf072_supplementary_data.docx]

**Appendix A:**

**Table A1:** Comparison of Odds Ratio of Different Parameters among Waves of COVID-19 with Forest plot

|  | Wave 2  vs  Wave 3 | Wave 2  vs  Wave 4 | Wave 2  vs  Wave 5 | Wave 3  Vs  Wave 4 | Wave 3  Vs  Wave 5 | Wave 4  Vs  Wave 5 |
| --- | --- | --- | --- | --- | --- | --- |
| Parameter | Odds Ratio (CI) | Odds Ratio (CI) | Odds Ratio (CI) | Odds Ratio (CI) | Odds Ratio (CI) | Odds Ratio (CI) |
| Male | 0.93(0.77-1.1) | 1.17(1.1-1.3) | 1.33(0.53-1.7) | 1.57(1.3-1.9) | 2.23(1.75-2.85) | 1.42(1.12-1.8) |
| Discharged | 1.80(0.73-2.2) | 3.18(2.6- 3.9) | 2.71(2.1-3.45) | 1.77(1.4-2.18) | 1.51(1.17-1.93) | 0.85(0.67-1.1) |
| Expired | 0.55(0.45-0.7) | 0.31(0.3-0.4) | 0.37(0.29-0.5) | 0.57(0.46-0.69) | 0.66(0.52-0.85) | 1.18(0.9-1.5) |
| Comorbidity | 1.44(1.2-1.8) | 1.4(1.18 - 1.8) | 0.57(0.43-0.8) | 1.003(0.8-1.24) | 0.39(0.29-0.54) | 0.4(0.29-0.54) |
| Diabetes Mellitus (DM) | 0.34(0.28-0.4) | 0.77(0.7-0.9) | 0.62(0.49-0.7) | 1.11(0.9-1.35) | 0.88(0.69-1.11) | 0.79(0.6-1.01) |
| Hypertension (HTN) | 0.62(0.52-75) | 0.63(0.5- 0.7) | 0.36(0.29-0.5) | 1.02(0.84-1.24) | 0.58(0.46-0.74) | 0.57(0.45-0.7) |
| Smoker | 0.68(0.30-1.5) | 1.33(0.5-3.4) | 0.22 (0.11-0.5) | 1.95(0.77-5.05) | 0.33(0.16-0.68) | 0.17(0.07-0.4) |
| Asthma | 1.25(0.73-2.2) | 1.20(0.7-2.1) | 0.66(0.37-1.1) | 0.95(0.5-1.75) | 0.52(0.28-0.98) | 0.54(0.29-1.0) |
| Ischemic Heart Disease (IHD) | 0.74(0.6-0.97) | 1.4(0.99-1.8) | 0.55 (0.41-0.8) | 1.85(1.3-2.55) | 0.76(0.55-1.05) | 0.41(0.3-0.59) |
| Cerebral Vascular Accident (CVA) | 0.95(0.45-1.5) | 1.5(0.66-3.3) | 0.24 (0.13-0.4) | 1.58(0.65-3.64) | 0.25(0.13-0.50) | 0.16(0.08-0.4) |
| Chronic Kidney Disease (CKD) | 1.62(0.88-2.9) | 1.2(0.7-2.3) | 0.34 (0.2-0.55) | 1.33(0.7-2.42) | 0.36(0.21-0.61) | 0.27(0.15-0.5) |
| Malignancy | 3.45(0.8-15.8) | 1.3(0.85-3.5) | 0.69(0.26-1.8) | 0.39(0.08-1.78) | 0.20(0.04-0.94) | 0.53(0.17-1.6) |
| Thyroid Abnormality | 0.8(0.42-1.46) | 0.7(0.37-1.3) | 0.4(0.21-0.7) | 0.87(0.46-1.69) | 0.05 (0.26-0.97) | 0.57(0.30-1.1) |
| Chronic Obstructive Pulmonary Disease (COPD) | 0.79(0.37-1.6) | 3.3(1.05-11) | 0.34 (0.17-0.7) | 4.24(1.3-14.03) | 0.44(0.21-0.93) | 0.1(0.03-0.33) |
| Others | 1.7(1.29-2.3) | 1.75(1.3-2.3) | 0.59(0.45-0.7) | 1.02(0.74-1.4) | 0.35(0.25-0.47) | 0.34(0.25-0.5) |
| Mechanical Ventilation | 1.23(0.97-1.5) | 0.56(0.5-0.7) | 1.34(0.98-1.8) | 0.7(0.54-0.87) | 1.64(1.17-2.29) | 2.38(1.7-3.28) |
| Inotropes | 1.35(1.07-1.7) | 0.52(0.4-0.6) | 0.65(0.49-0.8) | 0.70 (0.56-0.88) | 0.87(0.66-1.16) | 1.25(0.95-1.7) |
| Cytokine release syndrome (CRS) | 0.8(0.67-0.98) | 0.8(0.7-1.02) | 1.94(1.46-2.6) | 1.04(0.84-1.28) | 2.39(1.78-3.22) | 2.31(1.7-3.1) |
| Intensive Care Unit (ICU) Stay | 1.04(0.9-1.3) | 0.76(0.6-0.9) | 0.81(0.64-1.0) | 0.8(0.65-0.97) | 0.84(0.66-1.07) | 1.06(0.8-1.36) |
| Disease category at the time of admission |  |  |  |  |  |  |
| Mild COVID | 1.19(0.92-1.5) | 1.97(1.5-2.6) | 0.66 (0.50-0.9) | 1.66(1.21-2.27) | 0.56(0.41-0.76) | 0.33(0.24-0.5) |
| Moderate COVID | 0.81(0.62-1.1) | 2.01(1.4-2.8) | 2.77(1.68-4.6) | 2.49(1.75-3.58) | 3.43(2.08-5.67) | 1.38(0.79-2.4) |
| Severe COVID | 1.05(0.87-1.3) | 0.53(0.4-0.6) | 1.26(0.49-1.6) | 0.51 (0.41-0.6) | 1.2(0.93-1.53) | 2.35(1.81-3.1) |
| Critical COVID | 0.85(0.58-1.2) | 0.9(0.64-1.4) | 0.46(0.3-0.69) | 1.09(0.73-1.64) | 0.55(0.37-0.8) | 0.50(0.3-0.77) |
|  |  |  |  |  |  |  |
| Complications | 1.36(1.14-1.6) | 0.5(0.42-0.6) | 0.49 (0.4-0.63) | 0.69 (0.56-0.83) | 0.68(0.53-0.86) | 0.99(0.77-1.3) |
| Adverse Respiratory Distress Syndrome (ARDS) | 1.03(0.85-1.2) | 0.7(0.54-0.7) | 1.01(0.79-1.3) | 0.68 (0.55-0.82) | 1.04(0.81-1.35) | 1.55(1.2-1.99) |
| Pneumonia | 1.69(1.4-2.06) | 0.35(0.3-0.4) | 0.44(0.4-0.56) | 0.6(0.49-0.73) | 0.75(0.59-0.95) | 1.25(0.99-1.6) |
| Non-ST-Elevation Myocardial Infarction(NSTEMI) | 1.16(0.85-1.6) | 0.5(0.3-0.63) | 0.45(0.3-0.63) | 0.5 (0.37-0.74) | 0.52(0.37-0.74) | 0.79(0.57-1.1) |
| Septic Shock | 1.57(1.24-1.9) | 0.5(0.4-0.65) | 0.74(0.54-0.9) | 0.8 (0.64-1.022) | 1.15(0.85-1.55) | 1.43(1.06-1.9) |
| Pulmonary Embolism (PE) | 1.43(1.01-2.0) | 0.64(0.5-0.9) | 0.74(0.47-1.1) | 0.9(0.65-1.314) | 1.06(0.69-1.64) | 1.06(0.69-1.6) |
| Acute Kidney Injury(AKI) | 1.6(1.28-2.0) | 0.5(0.42-0.7) | 0.41(0.32-0.5) | 0.86 (0.68-1.1) | 0.66 (0.50-0.87) | 0.77(0.6-1.01) |
| Pneumothorax | 0.85(0.33-2.2) | 0.6(0.26-1.3) | 1.40(0.4-4.7) | 0.5(0.19-1.32) | 1.2(0.34-4.28) | 2.3(0.68-7.7) |
| Subcutaneous Emphysema | 0.8(0.37-1.8) | 1.3(0.61-3.0) | 6.4(1.15-67.3) | 1.08(0.44-2.6) | 5.18(0.90-56.46) | 6.99(1.1-74.7) |
| Disease progression | 1.22(1.01-1.5) | 0.59(0.5-0.7) | 0.75(0.6-0.9) | 0.73(0.59-0.88) | 0.92(0.73-1.17) | 1.27(1.01-1.6) |

**A 2: Comparison of Clinical Profile of Patients with COVID in Wave 4 and 5 using Binary Regression**

| **Category** | **Wave 4**  **n=783 (100%)** | | | | | **Wave 5**  **n=415 (100%)** | | | | |
| --- | --- | --- | --- | --- | --- | --- | --- | --- | --- | --- |
|  | **Survivor n=450 (100%)** | **Non-Survivor n=333 (100%)** | ***P*-value** | **Multivariable**  **AOR^*^ (95% CI)** | ***P*-value** | **Survivor**  **n=254 (100%)** | **Non-Survivor n=161 (100%)** | ***P*-value** | **Multivariable**  **AOR^*^ (95% CI)** | ***P*-value** |
| **Age group (years)** |  |  |  |  |  |  |  |  |  |  |
| **16-35** | 43 (9.6) | 6 (1.8) | <0.0001 | 1.0 |  | 18 (7.1) | 1 (0.1) | <0.0001 | 1.0 |  |
| **36-55** | 176 (39) | 90 (27) |  | 4.10 (1.6-10.3) | 0.003 | 48 (18.9) | 18 (11.2) |  | 6.3 (0.74-52.9) | 0.091 |
| **56-75** | 202 (45) | 189 (56.8) |  | 7.9 (3.2-19.5) | <0.0001 | 136 (53.5) | 86 (53.4) |  | 11.1 (1.4-88.4) | 0.23 |
| **>75** | 29 (6.4) | 48 (14.4) |  | 14.3 (5.2-39.1) | <0.0001 | 52 (20.5) | 56 (34.8) |  | 21.4 (2.6-176) | 0.004 |
| **Male** | 238 (53) | 179 (53.8) | 0.828 | 1.17 (0.4-3.1) | 0.753 | 137 (53.9) | 93 (57.8) | 0.479 | 1.0 (0.66-1.61) | 0.892 |
| **Female** | 212 (47) | 154 (46.2) |  | 1.0 |  | 117 (46.1) | 68 (42.2) |  | 1.0 |  |
| **Comorbidities** | 303 (67) | 234 (70.3) | 0.393 | 1.0 (0.6-1.64) | 0.987 | 218 (85.8) | 133 (82.6) | 0.404 | 1.18 (0.56-2.5) | 0.669 |
| **Diabetes mellitus** | 173 (38) | 128 (38.4) | 1 | 1.05 (0.72-1.5) | 0.82 | 118 (46.5) | 65 (40.4) | 0.264 | 1.32 (0.81-2.15) | 0.269 |
| **Hypertension** | 217 (48) | 167 (50.2) | 0.613 | 1.1 (0.73-1.68) | 0.987 | 161 (63.4) | 99 (61.5) | 0.755 | 1.09 (0.6-1.87) | 0.761 |
| **Smoker** | 2 (0.4) | 4 (1.2) | 0.41 | 2.5 (0.3-19.0) | 0.361 | 11 (4.3) | 7 (4.3) | 1 | 1.26 (0.41-3.89) | 0.686 |
| **Asthma** | 9 (2) | 12 (3.6) | 0.185 | 1.54 (0.58-4.1) | 0.39 | 13 (5.1) | 7 (4.3) | 0.817 | 1.36 (0.49-3.7) | 0.556 |
| **Ischemic Heart Disease** | 31 (6.9) | 31 (9.3) |  | 1.52 (0.9-2.7) | 0.159 | 38 (15) | 34 (21.1) | 0.112 | 1.44 (0.81-2.56) | 0.221 |
| **Cerebral Vascular Accident** | 6 (1.3 ) | 2 (0.6) | 0.478 | 2.9 (0.5-16.5) | 0.207 | 9 (3.5) | 16 (9.9) | 0.01 | 2.9 (1.2-7.4) | 0.019 |
| **Chronic Kidney Disease** | 10 (2.2) | 8 (2.4) | 1 | 1.69 (0.6-4.75) | 0.325 | 22 (8.7) | 11 (6.8) | 0.579 | 1.06 (0.46-2.5) | 0.892 |
| **Malignancy** | 3 (0.7) | 2 (0.6) | 1 | 1.29 (0.2-8.77) | 0.794 | 4 (1.6) | 1 (0.6) | 0.653 | 2.05 (0.2-21.09) | 0.546 |
| **Thyroid abnormality** | 9 (2) | 11 (3.3) | 0.262 | 1.17 (0.4-3.1) | 0.753 | 11 (4.3) | 7 (4.3) | 1 | 1.04 (0.35-3.05) | 0.942 |
| **Disease Progression** | 211 (47) | 187 (56.2) | 0.011 | 1.4 (0.90-2.17) | 0.126 | 107 (42.1) | 79 (49.1) | 0.188 | 1.75 (0.97-3.16) | 0.064 |
| **Unvaccinated** | 360 (80) | 284 (85.3) | 0.056 | 1.0 | 0.061 | 166 (65.4) | 119 (73.9) | 0.082 | 1.0 |  |
| **Vaccinated** | 90 (20) | 49 (14.7) |  | 0.68 (0.45-1.02) |  | 88 (34.6) | 42 (26.1) |  | 0.65 (0.4-1.06) | 0.075 |
| **ICU Stay** | 178 (39) | 154 (46.4) | 0.061 | 1.18 (0.68-2.02) | 0.555 | 103 (40.6) | 67 (41.6) | 0.838 | 1.2 (0.61-2.39) | 0.596 |
| **Complications** |  |  |  |  |  |  |  |  |  |  |
| **Acute Respiratory Distress Syndrome** | 182 (40) | 145 (43.5) | 0.42 | 1.12 (0.72-1.72) | 0.623 | 83 (32.7) | 48 (29.8) | 0.58 | 1.92 (1.0-3.62) | 0.045 |
| **Pneumonia** | 201 (45) | 171 (51.4) | 0.07 | 1.09 (0.74-1.60 | 0.671 | 104 (40.9) | 70 (43.5) | 0.612 | 1.4 (0.814-2.48) | 0.217 |
| **Myocardial Infarction** | 58 (12.9) | 52 (15.6) | 0.299 | 1.05 (0.65-1.7) | 0.837 | 45 (17.7) | 26 (16.1) |  | 1.36 (0.7-2.64) | 0.364 |
| **Septic Shock** | 102 (23) | 88 (26.4) | 0.238 | 1.052 (0.6-1.83) | 0.857 | 43 (16.9) | 33 (20.5) | 0.365 | 1.024 (0.456-2.3) | 0.954 |
| **Pulmonary Embolism** | 31 (6.9) | 38 (11.4) | 0.03 | 1.87 (1.06-3.3) | 0.03 | 24 (9.4) | 8 (5) | 0.13 | 2.1 (0.83-5.38) | 0.117 |
| **Acute Kidney injury** | 104 (23) | 85 (25.5) | 0.448 | 1.23 (0.8-1.91) | 0.345 | 71 (28) | 50 (31.1) | 0.508 | 1.01 (0.59-1.73) | 0.982 |
| **Pneumothorax** | 3 (0.7) | 10 (3) | 0.02 | 4.31 (1.04-18) | 0.045 | 1 (0.4) | 2 (1.2) | 0.563 | 4.9 (0.32-75.27) | 0.252 |
| **Cytokine Release Syndrome** | 137 (30) | 113 (33.9) | 0.314 | 1.02 (0.7-1.45) | 0.936 | 45 (17.7) | 25 (15.5) | 0.593 | 1.42 (0.73-2.76) | 0.297 |
| **Mechanical Ventilation `** | 116 (26) | 96 (28.8) | 0.371 | 1.13 (0.65-1.97) | 0.674 | 33 (13) | 23 (14.3) | 0.769 | 1.15 (0.54-2.43) | 0.725 |
